# University-Based Outbreaks of Meningococcal Disease Caused by Serogroup B, United States, 2013–2018

**DOI:** 10.3201/eid2503.181574

**Published:** 2019-03

**Authors:** Heidi M. Soeters, Lucy A. McNamara, Amy E. Blain, Melissa Whaley, Jessica R. MacNeil, Susan Hariri, Sarah A. Mbaeyi, for the Serogroup B Meningococcal Disease University Outbreak Group

**Affiliations:** Centers for Disease Control and Prevention, Atlanta, Georgia, USA

**Keywords:** bacteria, bacterial infection, infectious disease, meningitis/encephalitis, meningococcal meningitis, meningococcal vaccines, serogroup B, universities, vaccination recommendations, United States, vaccines

## Abstract

We reviewed university-based outbreaks of meningococcal disease caused by serogroup B and vaccination responses in the United States in the years following serogroup B meningococcal (MenB) vaccine availability. Ten university-based outbreaks occurred in 7 states during 2013–2018, causing a total of 39 cases and 2 deaths. Outbreaks occurred at universities with 3,600–35,000 undergraduates. Outbreak case counts ranged from 2 to 9 cases; outbreak duration ranged from 0 to 376 days. All 10 universities implemented MenB vaccination: 3 primarily used MenB-FHbp and 7 used MenB-4C. Estimated first-dose vaccination coverage ranged from 14% to 98%. In 5 outbreaks, additional cases occurred 6–259 days following MenB vaccination initiation. Although it is difficult to predict outbreak trajectories and evaluate the effects of public health response measures, achieving high MenB vaccination coverage is crucial to help protect at-risk persons during outbreaks of meningococcal disease caused by this serogroup.

Meningococcal disease, caused by the bacterium *Neisseria meningitidis*, is a severe, life-threatening illness with rapid onset and progression of symptoms. Case-fatality rates can be as high as 10%–20% among treated persons (*1*); 11%–19% of survivors develop major clinical sequelae, including loss of limbs, deafness, and seizures (*2*). In the United States, meningococcal disease incidence has steadily declined since 1995 (1.20 cases/100,000 persons) to a historic low of 0.11 cases/100,000 persons in 2017 (*3*). 

Of the 4 meningococcal serogroups (B, C, W, Y) that cause most cases of the disease in the United States, serogroup B is currently the predominant serogroup overall and accounts for more than half of meningococcal disease cases among persons 16–20 years of age (*1*). Although the overall incidence is low, university students are at increased risk of meningococcal disease caused by serogroup B compared with other adolescents and young adults who do not attend university in the United States (*4*).

Vaccination is the primary strategy for prevention of meningococcal disease. Since 2005, the US Advisory Committee on Immunization Practices has recommended quadrivalent meningococcal conjugate vaccine covering serogroups A, C, W, and Y (MenACWY) for routine use in adolescents 11–18 years of age and other groups at increased risk for meningococcal disease, including unvaccinated college freshmen living in dormitories (*5*). In 2013, a serogroup B meningococcal (MenB) vaccine, MenB-4C (Bexsero; GlaxoSmithKline, https://www.gsk.com) (*6*), became available for outbreak response via a Centers for Disease Control and Prevention (CDC)–sponsored expanded access investigational new drug protocol. In 2014–2015, MenB-FHbp (Trumenba; Pfizer, https://www.pfizer.com) (*7*) and MenB-4C were licensed for use in the United States. Although these vaccines are not routinely recommended for all adolescents or college students, adolescents and adults 16–23 years of age may be vaccinated with a MenB series based on individual clinical decision-making (*8*). In addition, MenB vaccine is recommended for use in persons ≥10 years of age who are at increased risk for meningococcal disease caused by this serogroup, including during outbreaks (*9*). In outbreak settings, either a 2-dose series of MenB-4C (0, ≥1 month) or a 3-dose series of MenB-FHbp (0, 1–2, 6 months) is recommended (*10*).

Historically, most meningococcal disease outbreaks on university campuses in the United States were caused by serogroup C (*11*,*12*). However, serogroup B has caused all known US university-based outbreaks since 2011, likely in part because of high MenACWY coverage in adolescents (*13*). We summarize university-based outbreaks of meningococcal disease caused by serogroup B in the United States in the years following MenB vaccine availability (2013–2018) and describe the resulting MenB vaccination responses.

## Identifying University-Based Outbreaks

Outbreaks of meningococcal disease among university students are usually reported to CDC by state health departments as part of routine technical assistance. We also reviewed cases of meningococcal disease reported through the National Notifiable Diseases Surveillance System, supplemented since 2015 with data collected through enhanced meningococcal disease surveillance activities to improve completion of key variables, including association with an outbreak. However, this review did not identify any other previously unreported meningococcal disease outbreaks caused by serogroup B in university students. This analysis includes all university-based outbreaks in which ≥2 cases of genetically related meningococcal disease cases caused by serogroup B were reported during a 3-month period (*14*).

During January 2013–May 2018, a total of 10 university-based meningococcal disease outbreaks caused by serogroup B were reported in 7 states; these outbreaks resulted in a total of 39 cases and 2 deaths (5%) (Table 1). The median patient age was 19 years; 62% were male. Syndrome information was available for 38 (97%) cases: 63% were meningitis (with or without bacteremia) and 37% were bacteremia without focus. Only 1 case occurred in a student who previously received MenB vaccine; this student had been vaccinated with 1 dose of MenB vaccine 6 days before disease onset (*22*). The remaining cases occurred in persons believed to be unvaccinated with MenB vaccine. Thirty-six (92%) cases were in undergraduate students from 4-year degree-granting universities; 3 cases occurred in unvaccinated close contacts (*5*) of undergraduate students (*15*,*23*). In the 2017 Pennsylvania outbreak, the 2 cases were in close contacts on the same athletic team. In the remaining outbreaks, no direct epidemiologic links were identified between cases.

**Table 1:** University-based outbreaks of meningococcal disease caused by serogroup B, United States, 2013–2018

State of university	Outbreak period	No. cases (deaths)	Approximate no. undergraduates	Clonal complex of outbreak strain	Sequence type of outbreak strain	References
New Jersey	2013 Mar–2014 Mar	9 (1)*	5,000	41/44	409	(*15**,**16*)
California	2013 Nov	4†	19,000	32	32	(*17*)
Rhode Island	2015 Jan–Feb	2	3,700	Unassigned	9069	(*18**–**21*)
Oregon	2015 Jan–May	7 (1)‡	20,000	32	32	(*22**,**23*)
California	2016 Jan–Feb	2§	5,000	32	11910	(*24*)
New Jersey	2016 Mar–Apr	2	35,000	11	11	(*25*)
Wisconsin	2016 Oct	3	30,000	32	11556	(*26**,**27*)
Oregon	2016 Nov–2017 Nov	5	25,000	32	32	(*28*)
Massachusetts¶	2017 Oct–2018 Feb	3	26,000	41/44	41	(*29*)
Pennsylvania	2017 Nov	2#	3,600	32**	8758**	

*Two of the cases occurred in close contacts of undergraduate students at the outbreak-affected university: 1 in a high school student who stayed in an undergraduate dormitory and 1 in a student at a different university.  †One additional associated case that occurred in March 2013 was identified after retrospective case review. An additional case occurred 2 years later in a close contact of an undergraduate student who may have been connected to this outbreak. ‡One case occurred in a close contact of undergraduate students at the outbreak-affected university. §One additional suspected case with inconclusive lab results occurred in February 2016 in a student previously vaccinated with 2 doses of MenB vaccine and who received antimicrobial chemoprophylaxis the day before symptom onset. ¶Cases occurred at 2 universities in a college consortium in the same geographic area. The first 2 cases occurred at University 1 and the third case occurred at University 2. #Cases were close contacts and members of the same athletic team. **An isolate was available for only 1 of the 2 cases, so typing results refer to the single case isolate.

**Figure 1:**
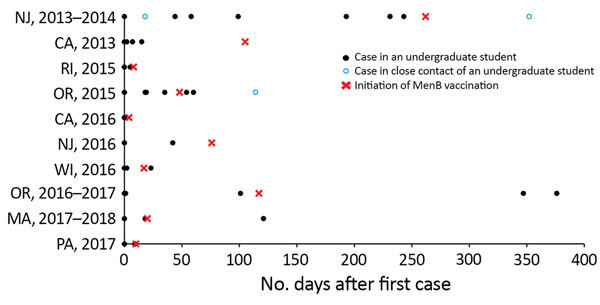
Timing of case onset dates and initiation of vaccination efforts during university-based outbreaks of meningococcal disease caused by serogroup B, United States, 2013–2018. MenB, serogroup B meningococcal vaccine.

Outbreaks occurred at universities ranging from small private schools with 3,600–5,000 undergraduates to large public universities with up to 35,000 undergraduates. In the 2017–2018 Massachusetts outbreak, cases occurred at 2 universities in a college consortium in the same geographic area. The number of cases per outbreak ranged from 2 to 9 (median 3 cases), and the attack rate ranged from 10 to 134 cases/100,000 population (median 35 cases/100,000 population). Outbreak duration (time from onset date of first to last case) ranged from 0 to 376 days (median 34 days) (Figure 1).

Six of 10 outbreaks were caused by strains of *N. meningitidis* belonging to clonal complex (CC) 32 (Table 1), a relatively common CC among serogroup B cases in the United States. Three outbreaks in Oregon and California were caused by *N. meningitidis* strains belonging to multilocus sequence type (ST) 32; the 2 Oregon outbreak strains had the same outer membrane protein types and vaccine antigen variants and clustered into a single clade, indicating that both outbreaks may have been derived from the same strain (*30*). The 3 other outbreak strains belonging to CC32 were ST11910 (2016 California), ST11556 (2016 Wisconsin), and ST8758 (2017 Pennsylvania). Two of the outbreaks in the northeastern United States were caused by strains belonging to CC41/44, another CC commonly associated with serogroup B in the United States, although each was caused by a distinct sequence type (ST409 in New Jersey and ST41 in Massachusetts). The remaining 2 outbreaks were caused by ST11/CC11 (2016 New Jersey), a relatively common sequence type but most often associated with serogroups C and W, and ST9069 (2015 Rhode Island), a rare strain not yet assigned to a clonal complex and not previously reported as a cause of invasive disease (*31*).

In all outbreaks, chemoprophylaxis was provided to close contacts of case-patients; in some cases, chemoprophylaxis recommendations were expanded to include those in common social networks, such as athletic teams or social organizations. None of the serogroup B cases occurred in students who had received chemoprophylaxis before symptom onset; 1 person received ciprofloxacin chemoprophylaxis after symptom onset but before diagnosis (*24*).

## MenB Vaccination Response

MenB vaccines were used in response to all 10 outbreaks (Table 2). Two of the outbreaks (New Jersey and California) occurred in 2013, before licensure of MenB vaccines; therefore, MenB-4C vaccine was provided through a CDC-sponsored expanded-access investigational new drug protocol (*15*,*17*). The remaining 8 outbreaks occurred following the licensure of MenB-4C and MenB-FHbp vaccines in the United States: 3 of the affected universities implemented MenB vaccination using primarily MenB-FHbp, and 5 universities primarily used MenB-4C. The choice of which MenB vaccine to use was driven largely by vaccine availability and procurement mechanisms at the time of the outbreak. In some instances, vaccine dosing schedule was also considered. In the 2016 New Jersey outbreak, a preferential recommendation was made for MenB-FHbp based on molecular and immunologic data (*25*).

**Table 2:** Vaccination response to university-based outbreaks of meningococcal disease caused by serogroup B, United States, 2013–2018*

State of university	Primary MenB vaccine	Vaccination strategy	Vaccination coverage	Cases after vaccine implementation
New Jersey	MenB-4C†	Mass vaccination campaign	Dose 1, 95%; dose 2, 89%	1
California	MenB-4C†	Mass vaccination campaign	Dose 1, 51%; dose 2, 40%	0
Rhode Island	MenB-FHbp	Mass vaccination campaign	Dose 1, 94%; dose 2, 80%; dose 3, 77%‡	0
Oregon	MenB-FHbp§	Mass vaccination campaigns; student health; local pharmacies	Dose 1, 52%; dose 2, 40%; dose 3, 10%	3¶
California	MenB-4C	Mass vaccination campaign	Dose 1, 90%; dose 2, 90%#	0
New Jersey	MenB-FHbp	Local providers/pharmacies; student health; some vaccination campaigns	NA	0
Wisconsin	MenB-4C	Mass vaccination campaign	Dose 1, 67%; dose 2, >31%**	1¶
Oregon	MenB-4C	Mass vaccination campaign with targeted student groups; vaccine requirement	Dose 1, 98%; dose 2, 93%	2
Massachusetts††	MenB-4C	Mass vaccination campaign; student health; providers/pharmacies	Dose 1, 34%**,‡‡; dose 2, 16%	1
Pennsylvania	MenB-4C	Vaccinated athletic team; campus-wide recommendation for vaccination at student health	Dose 1, 14%; dose 2, 2%**	0

**Figure 2:**
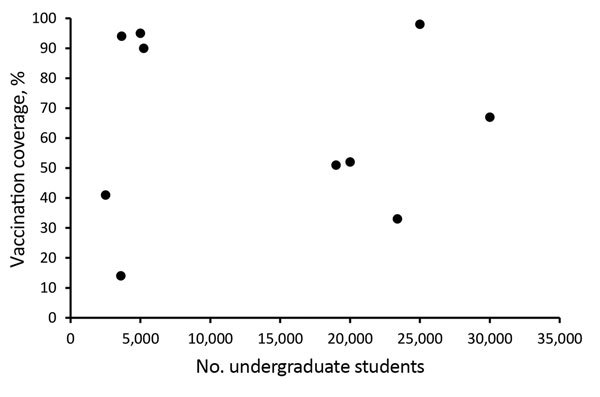
Association between university size and first-dose serogroup B meningococcal vaccine coverage in response to university-based outbreaks, United States, 2013–2018.

*MenB, serogroup B meningococcal; MenB-4C, Bexsero (GlaxoSmithKline, https://www.gsk.com); MenB-FHbp, Trumenba (Pfizer, https://www.pfizer.com); NA, vaccination coverage estimates not available. †Vaccine available through a CDC-sponsored investigational new drug protocol. ‡Coverage data refer to 2015 campaign. Incoming freshmen and returning study abroad students were also vaccinated in 2015–2016. §MenB-4C was also used in response to this outbreak; 21% of students completed either vaccine series. Presented coverage data for this university are as of July 2018. ¶One case in each of these outbreaks occurred 6 days after MenB vaccination efforts commenced, thus prior to any expected immunity. #This coverage estimate represents the number of persons vaccinated in the second campaign, which likely included some first doses. **Reported coverage reflects only vaccine doses given on campus; additional students received vaccine doses from providers or pharmacies at home or in other states. ††Cases occurred at 2 universities in a college consortium in the same geographic area. The first 2 cases occurred at university 1 and the third case occurred at university 2. Both universities began vaccination efforts following the second case. ‡‡Combined first-dose coverage data for both universities are presented in the table. First-dose coverage was 33% at university 1 (23,388 undergraduates) and 41% at university 2 (2,500 undergraduates) (Figure 2). Vaccination was ongoing at the time of publication; therefore, data should be considered preliminary.

MenB vaccination was implemented in a variety of ways. Mass vaccination campaigns were used in all but 2 outbreaks; most universities also made MenB vaccination available by appointment at the student health clinic. Some schools also encouraged students to seek vaccination through pharmacies and providers, 1 university integrated MenB vaccination into seasonal influenza vaccination clinics, and 2 universities instituted vaccination requirements.

Populations recommended for vaccination based on risk of exposure to the outbreak strain were similar at each university: all undergraduate students; graduate students living in undergraduate or graduate dormitories; and students, faculty, and staff with medical conditions putting them at increased risk for meningococcal disease. Some universities made additional efforts to target specific subpopulations of students for MenB vaccination, because of either the epidemiology of the cases or increased social mixing and close contact among specific student organizations, such as athletic teams or social organizations. Some universities also recommended vaccination for other small groups, such as spouses living with undergraduates in dormitories, persons in intimate physical relationships with undergraduates, and other staff or graduate students <25 years of age.

Even with mass vaccination campaigns, MenB vaccination coverage in response to university-based outbreaks was highly variable, ranging from an estimated 14% to 98% coverage for the first dose (median 67%) (Figure 2). In general, some small universities were able to achieve high coverage; however, 2 large universities with >25,000 undergraduates achieved high coverage as well. High first-dose coverage (67%) was achieved in response to the 2016 Wisconsin outbreak, driven by continued cases after vaccination efforts began and the use of federal funds to procure vaccine, thereby relieving hurdles related to vaccine financing and insurance coverage (*26*). In the 2016–2017 Oregon outbreak, an initial first-dose vaccination coverage of 8% was increased to 98% after continued cases prompted intensified vaccination efforts; eventually, the university opted to require proof of MenB vaccination as a prerequisite for registration (*28*). Vaccination efforts in response to the Massachusetts outbreak were ongoing at the time of publication, with coverage estimates expected to increase. In addition, several universities were able to track only vaccine doses administered on campus; because additional students received MenB vaccine from external providers and pharmacies, reported coverages are considered minimum estimates.

In the 2 outbreaks in 2013, MenB vaccine was provided through an expanded access investigational new drug protocol (*15*,*17*). In the 2013 California outbreak, a vaccination campaign was initiated after 4 cases had occurred; no additional cases occurred following MenB vaccination implementation (Figure 1). In the 2013–2014 New Jersey outbreak, vaccination was implemented after 8 cases had occurred; an additional case occurred 90 days after the initiation of the vaccination campaign in an unvaccinated close contact of an undergraduate student. Among the 8 outbreaks that occurred following MenB vaccine licensure in the United States, 6 universities implemented MenB vaccination after 2 cases, 1 after 3 cases, and 1 after 4 cases. In the 6 outbreaks in which vaccination was implemented after the first 2 cases, 4 had no further outbreak-associated cases and 2 had 1 additional case each: 1 occurred 6 days after the initiation of a MenB vaccination campaign (2016 Wisconsin), and 1 occurred 101 days after MenB vaccination implementation (2017–2018 Massachusetts) but occurred at a nearby university in the same college consortium that had already begun vaccination efforts. The university that implemented vaccination after the first 3 cases (2016–2017 Oregon) had 2 additional cases occur, 230 and 259 days after the initiation of MenB campaigns. The university that implemented vaccination after the first 4 cases (2015 Oregon) had 3 additional cases occur, 6, 12, and 66 days after campaign initiation; only the case that occurred 6 days after campaign initiation was in a person who had been vaccinated (*22*). In total, 8 additional cases occurred after the initiation of MenB vaccination campaigns in 5 outbreaks: 3 cases within 2 weeks and 5 cases in 66–259 days (median 101 days), although 3 of these later cases occurred in persons outside the original university target population for vaccination.

In the United States, for the purposes of public health decision making, the risk of meningococcal disease may be considered to have returned to expected levels 1 year following the last case in a university-based outbreak (*14*). For this reason, or in response to additional cases, MenB vaccination efforts continued into the following school year at some universities, whether in the form of mass campaigns, offering MenB vaccine at student health, or continuing or instituting MenB vaccination recommendations or requirements.

## Discussion

Although the incidence of meningococcal disease in the United States is low, university students are at increased risk of meningococcal disease caused by serogroup B and outbreaks (*4*). In 2013–2018, from 1 to 4 university-based outbreaks occurred annually. These outbreaks varied in size and duration and affected both small and large universities. All the outbreaks occurred at residential 4-year degree-granting universities. Similar university-based disease outbreaks were reported in Canada in 2015 (2 cases, 1 death) (*32*) and the United Kingdom in 2017 (3 cases, 1 death) (*33*).

Although MenB vaccination coverage among all US adolescents and young adults is unknown, coverage with >1 dose of MenB vaccine among persons 17 years of age was estimated to be 14.5% in 2017 (*13*), and a recent survey demonstrated that only 2% of universities require MenB vaccination for students (*34*). With a mostly unvaccinated student population, most outbreak-affected universities implemented mass MenB vaccination campaigns to quickly increase vaccination coverage, requiring an immense mobilization of public health resources from the university, local and state health departments, and often CDC. Achieving high MenB vaccination coverage through mass vaccination campaigns during outbreaks has been most challenging for large universities, although 1 university achieved nearly 100% coverage after implementation and enforcement of a MenB vaccine requirement (2016–2017 Oregon).

In 2017, partly on the basis of the experiences of these recent university outbreaks, CDC revised its guidance for the evaluation and public health management of suspected outbreaks of meningococcal disease (*14*). In the revised guidance, the outbreak threshold for vaccine decision-making in an organization-based outbreak, such as at a university, is defined as 2–3 outbreak-associated cases within an organization during a 3-month period: “In most situations, 2 cases within an organization constitute an outbreak. However, in some situations, such as an outbreak within a large university (e.g., >20,000 undergraduate students) where no identifiable subgroup at risk within the population can be identified, it may be reasonable to declare an outbreak after 3 cases” (*14*). Decisions about whether, when, and how to offer MenB vaccine during an outbreak remain challenging and should be tailored to the unique epidemiology of each outbreak. At the same time as vaccination discussions and mobilization efforts begin, appropriate chemoprophylaxis, enhanced case detection, and communication efforts should be promptly initiated.

When dealing with university-based outbreaks, it is difficult to predict if or when additional cases may occur. In some outbreaks, cases occurred over a prolonged period of time, as in a 2008–2010 Ohio outbreak (*35*) (before MenB vaccine availability in the United States) and the 2013–2014 New Jersey outbreak (*15*). Half of the 10 studied outbreaks ended following implementation of MenB vaccination efforts, but additional cases occurred at the other 5 universities, mainly among unvaccinated students or close contacts. Factors that might contribute to the occurrence of new cases following implementation of a vaccination campaign include low vaccination coverage, resulting in a large number of students without direct vaccine protection, and lack of MenB vaccine impact on meningococcal carriage, precluding development of herd protection (*18**,**23*).

It is difficult to determine whether university-based outbreaks have increased in frequency in recent years, as reporting of outbreaks to CDC has historically been incomplete and routine national meningococcal disease surveillance began collecting standardized data on university attendance only in 2015. Enhanced detection and reporting, improved molecular methods to confirm strain relatedness, the recent availability of MenB vaccines for outbreak response, and a low and declining overall disease incidence leading to greater awareness of outbreaks may have contributed to the increase in reported outbreaks among university students. Three university-based outbreaks were reported in the United States during 2008–2011 (*35*); however, the lack of routinely collected information on historical meningococcal disease outbreaks limits comparisons of outbreak size or duration before and after MenB vaccine availability.

Achieving high MenB vaccination coverage is necessary to help protect persons during outbreaks of meningococcal disease caused by serogroup B. Efforts are under way to better understand MenB vaccine effectiveness and duration of protection, MenB vaccine strain coverage, and current risk factors for meningococcal disease among university students. This information, along with lessons learned when implementing MenB vaccination campaigns, could help guide and improve responses to future outbreaks.
